# U-SHAPED ASSOCIATION OF Α-KLOTHO WITH ALL AND CAUSE-SPECIFIC MORTALITY IN US ADULTS: A SECOND ANALYSIS OF NHANES

**DOI:** 10.1093/geroni/igad104.2789

**Published:** 2023-12-21

## Abstract

Laboratory studies have shown that mice deficient in the klotho gene have a shortened lifespan, but the epidemiological evidence linking circulating α-klotho to mortality is inconsistent. Our aim was to clarify the association between serum α-klotho and mortality using a representative population. From the National Health and Nutrition Examination Survey (2007-2016), 13748 participants aged 40-79 years with serum α-klotho test data were selected and followed up to 31 December 2019. Participants were equally divided into 5 groups (Q1 < 622.8pg/ml, Q2: 622.8-739.1pg/ml, Q3: 739.1-857.7pg/ml, Q4: 857.7-1033.1pg/ml, Q5 >1033.1pg/ml) based on quantiles of serum α-klotho concentrations, with the Q3 serving as the reference group. Multivariate Cox proportional hazards regression analysis showed that both participants with low and high serum α-klotho levels had a higher risk of all-cause mortality, compared to the reference group (HR[95%CI], Q1:1.38[1.13,1.69], Q5:HR 1.23[1.02,1.47]). We further found a non-linear and U-shaped association between α-klotho and all-cause, cancer-cause, and diabetes-cause mortality, but an L-shaped association between α-klotho and CVD-cause mortality by using cubic spline function model and smooth curve fitting (penalized spline method). We observed a significant negative association between α-klotho (log-transformed) and all-cause mortality (HR[95%CI], 0.70[0.55,0.91]) in the subgroup of participants with normal liver function. In conclusion, the U-shaped association was found between serum α-klotho and all-cause mortality among US adults, and the reasons for the increased risk of death associated with high levels of α-klotho require further investigation.

